# Effect of fire needle for ganglion cysts: A protocol of systematic review and meta-analysis of randomized controlled trials

**DOI:** 10.1097/MD.0000000000022602

**Published:** 2020-10-09

**Authors:** Jun Chen, Qifang Liu, Jun Xiong, Lunbin Lu, Siyuan Zhu, Zhiying Zhong, Genhua Tang, Xingchen Zhou, Han Guo, Zhijun Chen

**Affiliations:** a Jiangxi University of Traditional Chinese Medicine; b Nanchang First Hospital; c The Affiliated Hospital of Jiangxi University of Traditional Chinese Medicine, Nanchang, China.

**Keywords:** acupuncture, fire needle, ganglion cysts, protocol, systematic review, tendon sheath

## Abstract

**Background:**

Ganglion cysts (GCs) are tumor-like lesions that often occur in the soft tissues, which are mostly caused by the degeneration of mucin produced by the joint capsule and tendon sheath on the carpal dorsal joints of extremities. GCs may appear asymptomatic as benign tumors, but some patients also seek treatment because of the pain caused by these fluid-filled cysts. As a kind of complementary and alternative therapy, there have been some studies published in China which have proved that the fire needle has a better therapeutic effect on ganglion cyst. The purpose of this systematic review is to evaluate the efficacy of fire needle in the treatment of GCs.

**Methods:**

PubMed, EMBASE, the Cochrane Library, Chinese National Knowledge Infrastructure, Chinese VIP Information, Wanfang Database, and Chinese Biomedical Literature Database were searched by 2 reviewers from the inception until August 2020. The original study that randomised control trials of fire needle for GCs will be selected and is not limited by country or language. In addition, researches in progress, the reference lists and the citation lists of identified publications will be retrieved similarly. Study selection, data extraction, and assessment of the quality will be performed independently by 2 reviewers who have been trained prior to data extraction. A meta-analysis will be conduct if the quantity and quality of the original studies included are satisfactory; otherwise, a descriptive analysis will be conducted. Review Manager V5.4: (The Nordic Cochrane Centre, The Cochrane Collaboration, Copenhagen, Denmark) software will be using for data synthesis and assessment the risk of bias according by Cochrane Handbook.

**Result:**

This study will provide a comprehensive review of current evidence for the treatment of fire needle on GCs.

**Conclusion:**

The conclusion of this study will provide a judging basis that whether the treatment of GCs with fire needle is effective.

**INPLASY registration number:**

INPLASY202080032

## 1 Introduction

Ganglion cysts (GCs) is a cystic tumor-like lesion with pain as the most common symptom. Spherical masses can be observed at the body's surface, which is delimited by dense connective tissue and filled with gelatinous fluid that made up of glucosamine, albumen, globulin, and a high concentration of hyaluronic acid and the patient's pain of hand or wrist is mainly caused by these fluid-filled cysts.^[1]^ GCs can arise from either a joint or a tendon sheath but commonly occurs on the joints of the limbs. Most GCs occur in the wrist. Dorsal wrist GCs account for 60% to 70% of all GCs, with volar wrist GCs accounting for about 18% to 20%. GCs typically consist of a cyst sac and the cyst may have a single cavity or multiloculated.^[2]^ Under B-ultrasound, the cyst wall is thin and smooth, with clear boundary, showing medium or high echo, and the interior shows low echo shadow.^[3]^ GCs are not considered true cysts because they lack a cellular epithelial lining, seen in synovial tissue or adventitial bursa.^[4]^

The pathogenesis of GCs is not clear, but it is usually not caused by a single factor. The cause of its occurrence has included congenital anomaly already, also be the stimulation that receives local stress possibly.^[2]^ So there is no definitive treatment for the pathogeny. In order to relieve the patient's pain, many treatments have been adopted, non-operative treatment includes supportive splinting, non-steroidal anti-inflammatory drugs and aspiration,^[5,6]^ and surgical procedures, etc.^[7]^ These therapies are successfully employed in clinical practice for GCs treatment, but the treatment effects are not always Satisfactory with the long treatment cycle and high recurrence rate. Recurrence of the cyst following surgery has been reported to range from 4% to 40%.^[5]^ Therefore, in recent years, more and more studies tend to evaluate the efficacy and safety of complementary and alternative therapies in the treatment of GCs.

In China, acupuncture and moxibustion are effective traditional therapeutics and fire needle is an operation method in traditional acupuncture therapy. By stimulating the acupuncture points, the fire needle can dredge the meridians, accelerate the flow of qi and blood, and make the cyst dissipate.^[8,9]^ As an non-drug therapy, fire needle has been reported in some clinical studies that has certain curative effect on GCs. Therefore, the purpose of this study was to summarize the original research on the treatment of GCs with fire needle, so as to evaluate whether the treatment of GCs with fire needle is really effective.

## 2 Methods

### 2.1 Registration

This protocol will be reported according to the Preferred Reporting Items for Systematic Reviews and Meta-analyses Protocols.^[10]^ It is registered in the INPLASY (registration number, INPLASY202080032; https://inplasy.com/inplasy-2020-8-0032/).

### 2.2 Inclusion criteria for this overview

PICOS will be applied, including Population, Intervention, Comparison, Outcome, and Study.

#### 2.2.1 Types of studies

Randomized controlled trials (RCTs) with fire needle as the primary intervention for GCs will be included, and other studies such as case reports, and reviews will be excluded. No restrictions on country but language will be limited on English and Chinese.

#### 2.2.2 Types of participants

Participants diagnosed as GCs by clinicians referring to the New Routine for Diagnosis and Treatment^[11]^ will be included. No restrictions on gender, age, race, etc.

#### 2.2.3 Types of interventions

Without limits on course and dose, we will include studies in which fire needle is the primary intervention and, if necessary, we will include studies in which fire needle is combined with other active treatments versus active treatment alone.

#### 2.2.4 Types of comparisons

The selected RCTs should testify that the interventions were compared with a control group composed of placebo, sham acupuncture, no treatment, or other active therapies.

#### 2.2.5 Outcomes

Primary outcome: effective rate and the cyst diameter.

Secondary outcomes: recurrence rate; adverse events incidence caused by fire needle, such as dizziness, vomiting, weariness, etc.

### 2.3 Search methods for study identification

#### 2.3.1 Electronic searches

Two investigators will retrieve the relevant RCTs in the following databases: PubMed, Embase, the Cochrane Library, CNKI, Chinese VIP information, Wanfang Database, and CBM, from inception until August 2020 without restriction to publication status and languages. A comprehensive search strategy will be conducted, various combinations of MeSH items and free words will be searched synchronously, including “ganglion cysts”, “Tendon sheath cyst”, “fire needle”, “huo zhen” and etc. The preliminary search strategy for PubMed is presented in Table 1.

**Table 1:**
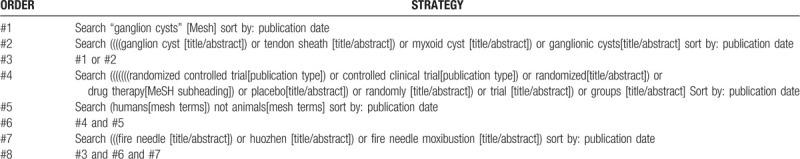
Search strategy for PubMed.

#### 2.3.2 Searching other resources

The relevant published references and citation list will be retrieved in Web of Science. In addition, the relevant systematic reviews or overview will also be identified for additional relevant studies. Moreover, relevant paper versions of medical journals and journals will be screened to ensure that the original studies that not included in the electronic databases could be included possibly.

### 2.4 Data collection and analysis

#### 2.4.1 Study selection

**Figure 1:**
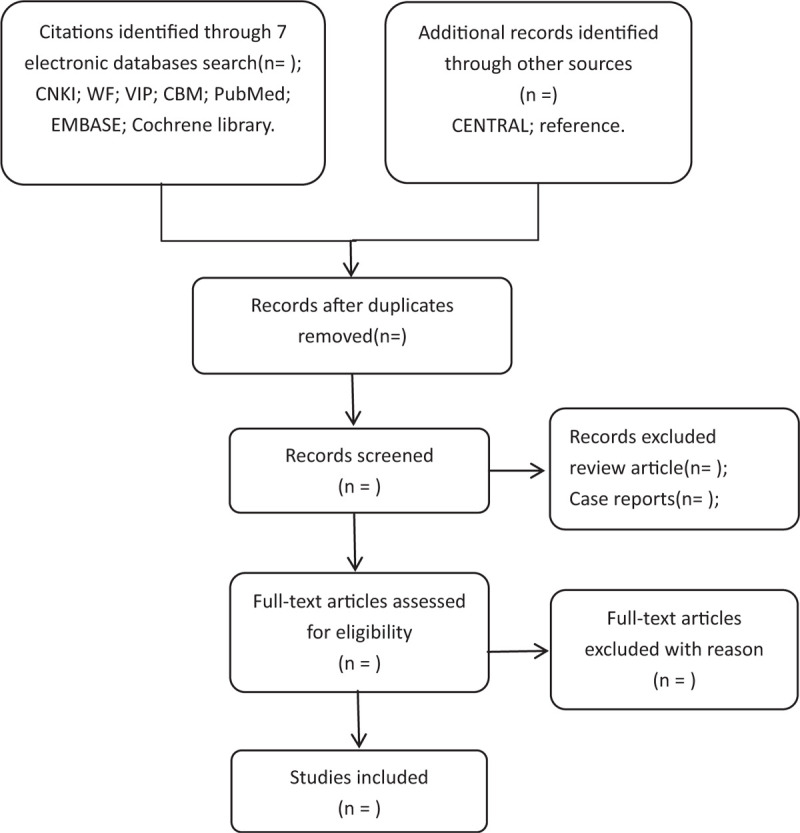
Flowchart of literature selection.

All reviewers undergo rigorous training prior to selecting the study. Preliminary screening of the study will be conducted by 2 reviewers independently. After searching, the duplicated studies will be removal initially from the retrieved studies by Endnote (X9). And then, 2 independent reviewers (JC and LBL) will screened titles, abstracts, and keywords of all retrieved studies for candidates according to the inclusion and exclusion criteria, we will obtain the full text of all possibly relevant studies. Excluded studies will be recorded with explanations. If it is uncertain whether to adopt because of the lack of information, LBL will try to contact authors of the original reports to obtain the information of lost. During the procedure, disagreements will be resolved by discussion or consensus with the third reviewer (JX). Study selection will be performed in accordance with the Preferred Reporting Items for Systematic Reviews and Meta-analyses flowchart (Fig. 1).

#### 2.4.2 Data extraction and management

A unified data extraction table will be designed before data extraction, and data extraction will also be carried out independently by 2 reviewers (SYZ and GHT). The proposed extracted information includes:

(1) General information: author, country, year of publication, study design, and database;
(2) Population characteristics: sex, age, baseline diseases, and sample size;
(3) methodological characteristics: information sources, intervention(s), comparison(s), bias assessment, etc. Any objections will be discussed by 2 reviewers, and further objections will be arbitrated by the third author (ZYZ).

#### 2.4.3 Assessment of risk of bias

To systematically evaluate the quality of each of the studies that final included. Two reviewers (SYZ and LBL) will assess the risk of bias for each included study according to the Cochrane handbook. It will eventually be rated on 3 levels (“high risk of bias”, “medium risk of bias” and “low risk of bias”).^[12]^ The specific evaluation items include the following 7 aspects: generation of random sequence, allocation concealment, blindness of participants, and personnel, blindness of outcome assessment, incomplete outcome data, selective reporting and other bias.

#### 2.4.4 Measures of treatment effect

Review Manager (RevMan V 5.4) will be used for data analysis and quantitative data synthesis. We will use the weight mean difference and 95% confidence interval to measure the continuous variables, while the results of dichotomous variables will using risk ratio and its 95% confidence interval.

#### 2.4.5 Dealing with missing data

If the specific information we need to collect are not be reported, the reviewer (GHT) will attempt to contact the original author for relevant information by telephone or e-mail. If the required information is not available, it will be explained in the article. Then, the missing data will be assumed to be “missing at random” and “missing not at random” according to the Cochrane Handbook.^[13]^ For the data missing at random, the analysis will rely on existing data, while we will filling the missing data with replacement values and make a sensitivity analysis to examine the potential impact of missing information, if necessary.

#### 2.4.6 Assessment of heterogeneity

Heterogeneity refers to the difference between studies in the systematic review,^[14]^ and the value of I^2^ represents the heterogeneity after data synthesis. We will use I^2^ to assess statistical heterogeneity between trials. If the I^2^ < 50%, that indicates slight or no heterogeneity in the evidence of the combined results, while I^2^ ≥ 50%, it means studies with high heterogeneity. The fixed effects model will be adopted when the *P* > .1 and I^2^ < 50%, while apply the random effect if *P* < .1 and I^2^ ≥ 50%.

#### 2.4.7 Assessment of reporting bias

An assessment of the reported bias will be presented in the form of a funnel plot. If the points on both sides of the funnel plot are scattered and asymmetric, it is considered that there is a report bias and the reliability of this study is low. On the contrary, if the point distribution on both sides of the funnel plot is symmetrical, we believe that there is no or very low reporting bias, and the results of this study are reliable.

#### 2.4.8 Data synthesis and subgroup analysis

All analysis will be done through RevMan 5.4. According to heterogeneity assessment, mean difference or relative risk were calculated using fixed or random effects models. In addition, if the I^2^ obtained after data consolidation is greater than 50% and the *P* value is less than .1, sensitivity or subgroup analysis will be performed to exclude the source of heterogeneity. If the included original research data is insufficient for quantitative analysis, the review will only represent, and summarize the evidence.

#### 2.4.9 Sensitivity analysis

If the results show significant heterogeneity and the number of included studies is sufficient, sensitivity analysis will be performed to identify the quality and robustness of the meta-analysis result, which includes assessing the impact of sample size, methodological elements and the characteristic of research, and missing data.

#### 2.4.10 Grading the quality of evidence

The quality of evidence will be evaluated using the Grading of Recommendations Assessment, Development, and Evaluation.^[15]^ The quality of evidences will be rated on 4 levels (high, medium, low, or very low). Two reviewers (XCZ and HG) will conduct the assessment process separately and describe in detail the reasons for downgraded or upgraded outcomes affecting the quality of evidence to guarantee the reliability and transparency of results.

## 3 Discussion

GCs are the most common tumors of the hand. Although they rarely deteriorate,^[16]^ they often cause pain, leading to significant motor dysfunction in the affected joint, which in turn reduces the patient's quality of life. Because the pathogenesis is not clear, according to the existing treatment principles, drug treatment is mainly non-steroidal anti-inflammatory drugs injection, non-drug treatment is mainly puncture aspiration, and surgical treatment. However, drug therapy is often accompanied by certain side effects, and the postoperative recurrence rate is high. As a result, many patients are looking for easier and less harmful alternatives.

As an alternative therapy for external therapy, the fire needle has a history of nearly 3 thousand years in China. It can relieve pain, improve the blood circulation, stimulate metabolism of local tissue.^[17]^ In recent years, a certain amount of studies conducted in China have shown that compared with conventional puncture aspiration and steroid administration, fire needle has a higher cure rate for the treatment of GCs.

However, the efficacy of fire needles in treating GCs has been controversial due to the lack of evidence-based medicine, and some studies have reported that acupuncture may be a placebo effect. To date, there is no reliable comprehensive review of the treatment of GCs with fire needle. We conducted this study to assess the efficacy of fire needles in the treatment of GCs and to provide clinical staff with a reliable treatment regimen. In addition, through this study, it is believed that more and higher quality original studies will be designed and carried out to provide more accurate guidance for the treatment of GCs.

## Acknowledgments

The authors would like to thank the following people who either provided feedback on the protocol or supported the development of the methods: Jun Chen, Qifang Liu, Jun Xiong, Lunbin Lu, Genhua Tang, Siyuan Zhu, Zhiying Zhong, Xingchen Zhou, Han Guo, Zhijun Chen.

## Author contributions

All authors have read and approved the publication of the protocol.

**Conceptualization:** Jun Chen, Jun Xiong.

**Data curation:** Jun Chen, Lunbin Lu, Siyuan Zhu, Zhiying Zhong, Xingchen Zhou, Han Guo, Genhua Tang.

**Formal analysis:** Jun Chen, Lunbin Lu.

**Investigation:** Jun Xiong, Jun Chen.

**Methodology:** Jun Chen, Siyuan Zhu, Lunbin Lu.

**Software:** Genhua Tang, Zhiying Zhong.

**Supervision:** Jun Xiong, Zhijun Chen, Xingchen Zhou.

**Writing – original draft:** Jun Xiong, Jun Chen, Qifang Liu.

**Writing – review & editing:** Jun Xiong, Zhijun Chen, Lunbin Lu, Siyuan Zhu.

## Abbreviations

Abbreviations: GCs = ganglion cysts, RCTs = randomized controlled trials.
